# Increasing Complexity of Molecular Landscapes in Human Hematopoietic Stem and Progenitor Cells during Development and Aging

**DOI:** 10.3390/ijms23073675

**Published:** 2022-03-27

**Authors:** Suzanne M. Watt, Peng Hua, Irene Roberts

**Affiliations:** 1 Stem Cell Research, Nuffield Division of Clinical Laboratory Sciences, Radcliffe Department of Medicine, University of Oxford, Oxford OX3 9BQ, UK; 2 Myeloma Research Laboratory, Adelaide Medical School, Faculty of Health and Medical Sciences, University of Adelaide, North Terrace, Adelaide 5005, Australia; 3 Cancer Program, Precision Medicine Theme, South Australian Health and Medical Research Institute, Adelaide 5001, Australia; 4 State Key Laboratory of Reproductive Medicine, Nanjing Medical University, Nanjing 210029, China; calvinhuapeng@hotmail.com; 5 MRC Molecular Haematology Unit, MRC Weatherall Institute of Molecular Medicine, and NIHR Oxford Biomedical Research Centre Haematology Theme, Radcliffe Department of Medicine, University of Oxford, Oxford OX3 9DU, UK; irene.roberts@paediatrics.ox.ac.uk; 6 Department of Paediatrics and NIHR Oxford Biomedical Research Centre Haematology Theme, University of Oxford, Oxford OX3 9DU, UK

**Keywords:** aging, development, hematopoietic stem cells, single-cell transcriptomics, childhood leukemias, clonal hematopoiesis of indeterminate potential, metabolism, inflamm-aging

## Abstract

The past five decades have seen significant progress in our understanding of human hematopoiesis. This has in part been due to the unprecedented development of advanced technologies, which have allowed the identification and characterization of rare subsets of human hematopoietic stem and progenitor cells and their lineage trajectories from embryonic through to adult life. Additionally, surrogate in vitro and in vivo models, although not fully recapitulating human hematopoiesis, have spurred on these scientific advances. These approaches have heightened our knowledge of hematological disorders and diseases and have led to their improved diagnosis and therapies. Here, we review human hematopoiesis at each end of the age spectrum, during embryonic and fetal development and on aging, providing exemplars of recent progress in deciphering the increasingly complex cellular and molecular hematopoietic landscapes in health and disease. This review concludes by highlighting links between chronic inflammation and metabolic and epigenetic changes associated with aging and in the development of clonal hematopoiesis.

## 1. Introduction

Human development and aging are associated with significant changes in functional hematopoietic cell outputs from specific anatomical regions and hematopoietic tissues [1,2,3,4]. Importantly, a detailed knowledge of these changes under steady-state and perturbed conditions is critical to understanding hematological disorders and diseases that originate during embryonic, fetal and post-natal life [5,6,7,8,9,10].

Although temporally different and not an exact recapitulation, murine studies have often been used as a model for human hematopoiesis [1,2,11]. From this and other research, the concept of a layered hematopoietic system has emerged [2,12,13], with successive, but distinct, waves of hematopoietic stem cell (HSC)-independent hematopoietic progenitor cells (HPCs) originating from the yolk sac and embryo proper prior to the generation of definitive HSCs principally, although not exclusively, from the embryonic para-aortic splanchnopleural/aorta-gonad-mesonephros (P-Sp/AGM) region. Selective HPCs and HSCs then seed the fetal liver, where they mature and expand before HSCs colonize the fetal bone marrow, with bone marrow becoming the main source of HSCs after birth [1,2,3,7]. Nevertheless, significant challenges still exist in studying HSCs and lineage-restricted HPCs during human embryonic and fetal development and on aging. These include difficulties in accessing and manipulating human tissues (e.g., inducing dynamic perturbations to validate the importance of putative key pathways), the rarity and molecular and functional heterogeneity of enriched HSC and early progenitor cell pools, inherent human variability, the effects of ex vivo manipulations, the relevance of surrogate models to human hematopoietic stem and progenitor cell (HSPC) physiology and pathology and differentiating between intrinsic and extrinsic regulatory mechanisms that control human HSPC fate.

Here and with these inherent biases in mind, we review recent molecular advances in single-cell (sc) omics analyses of human HSPCs, set against huge historical efforts spanning more than five decades that have aimed to identify and characterize human HSCs, their lineage-committed progeny during development and aging, and that provide mechanistic insights. Additional sophisticated technological advances over these decades include the discovery of monoclonal antibodies, the development of flow cytometry and cell sorting, of enhanced in situ imaging and single-cell capture technologies for the immunophenotypic identification and isolation of specific human HSPC subsets, of single-cell barcoding, lineage tracing, fate mapping and gene editing, and of sophisticated gene regulatory and three-dimensional genome organizational analyses, coupled with surrogate models in vivo and/or in vitro to assess the function of HSCs and their progeny, or following transplantation into human recipients as exemplified in some of our own and other studies [13,14,15,16,17,18,19,20,21,22,23,24,25,26,27,28,29,30,31,32,33,34,35,36,37,38,39]. Not only have these approaches provided insights into human hematopoiesis during development and aging, but they have also identified significant heterogeneity in HSCs and their progeny, led to newer concepts of lineage commitment and differentiation, and contributed to an understanding of the cell of origin for hematological disorders and diseases. Examples of these are discussed in this review.

## 2. The Concept of a Layered Hematopoietic System

Murine studies have provided considerable insight into the changes in hematopoiesis during development and aging [13,40,41]. The proposed layered organization of the hematopoietic system sees overlapping waves of HPCs and HSCs tailored to meet the specific needs of the embryo and its development into adulthood [2,7,12,13].

The first wave of primitive hematopoiesis originates in the murine yolk sac from about E7 (7 days post coitus; dpc), thus giving rise to nucleated erythroid cells, macrophages and megakaryocytes [42,43,44,45,46]. This is followed by a second wave of yolk sac hematopoiesis, termed pro-definitive or transient definitive hematopoiesis [2,7,13]. This coincides with the emergence of multipotent erythro-myeloid progenitors (EMP) from hemogenic endothelium (HE) at approximately E8–8.5 [46,47,48,49,50,51,52,53,54,55,56]. Between E9.5–10.5, these EMPs seed the fetal liver, where they generate myeloid cells, including erythroid cells, macrophages and granulocytes, and potentially low numbers of innate immune cells [2,7,13,40,46,47,48,49,50,51,52,53,54,55,56,57,58].

The third wave of murine hematopoiesis originates in the AGM region of the embryo proper, with immature or pro-HSCs emerging from distinct HE before maturing into definitive long-term repopulating (LT) HSCs (via type I and II pre-HSC) by around E11.5 [2,13,46,47,49,51,58,59,60,61,62,63,64,65,66]. HSC activity has additionally been found in the murine vitelline and umbilical arteries, embryonic head, heart and placenta [2,13,40,49,57,58,60,61,67,68,69]. It has been reported that HSC-independent HPCs also arise for example from distinct HE or intra-aortic clusters in the yolk sac and P-Sp/AGM region of the embryo proper at approximately E9.5 [13], with multipotential, lymphoid or lympho-myeloid biased progenitors preceding or emerging simultaneously with pre-HSCs [70,71,72]. Expansion and differentiation of second- and third-wave HPCs and definitive HSCs occur in the murine fetal liver before HSC colonization of the fetal spleen and fetal bone marrow [73]. MMP-3 (multi-potent progenitors-3) and lesser numbers of HSCs have also been reported to originate from HE in murine fetal/young adult bone marrow [74], perhaps constituting a fourth wave of hematopoiesis. Gradually, between about 3–4 weeks of post-natal life, the murine fetal bone marrow HSCs switch to an adult bone marrow phenotype in terms of their metabolic state, cell cycle behavior, self-renewal potential, lineage output and repopulation kinetics [75,76,77,78,79].

The contribution of yolk sac EMPs versus HSCs to initial lymphoid development is a matter of some debate [47]. For example, while some NK cells appear to be yolk sac EMP-derived [80], arguments for yolk sac EMP-derived lymphoid–myeloid progenitors (LMPs) initially seeding the developing thymus [81,82] contrast with recent evidence for the first thymic progenitors, a bipotent T and innate lymphoid cell (T/ILC) subset that generates lymphoid tissue inducer (LTi) cells and invariant Vγ5+ cells, being HSC-derived [83,84]. There has also been considerable debate regarding the origin of the first murine B cells, viz. innate-like sIgM^+^CD11b^+^CD5^+^ B-1a B cells of the peritoneal and pleural cavities, and mucosa [7], and particularly if these arise in the extra-embryonic yolk sac independently of HSCs or in the subsequent wave of definitive hematopoiesis from LT HSCs [85,86,87,88,89,90]. The layered immune system hypothesis supports the view that B-1 and B-2 B cells arise from distinct progenitors that are generated at different developmental stages [12,87]. An LT HSC independent origin of B-1a B cells was indicated when single murine adult bone marrow or fetal liver (E15) Lin^-^Sca-1^+^Kit^+^ (LSK) CD150^+^CD48^–^ LT HSCs failed to regenerate B-1a lymphoid cells in adult murine transplant models, in which the adult microenvironment had been shown to be conducive to B-1a B cell reconstitution [91,92]. This view was also supported by lineage tracing in adult mice [93,94], the presence of B-1a lymphoid cells in HSC-deficient transgenic murine embryos [90], and the reconstitution of B-1a cells, but not conventional B-2 lymphoid cells which mediate canonical adaptive immunity, from murine E9 yolk sac and AGM HE [7,95,96]. Barcoding studies, in contrast, have suggested that LSK enriched murine fetal liver (E14.5) HSPCs give rise to B-1a and B-2 lymphoid cells and splenic granulocytes [97], although more highly enriched LSK CD150^+^CD48^–^FLT3^–^ single fetal liver LT HSC transplants only inefficiently reconstituted B-1a cells in adult mice. Further fate mapping studies defined a transient or developmentally restricted KSL CD50^lo/-^ fetal HSC subset that could give rise to B-1a lymphoid cells and that differed from the previously defined LSK CD150^+^ LT HSC subset described above, but with these two HSC subsets possessing differential abilities to persist into adulthood [85,91,98]. Clonal studies by Hadland et al. [99] examined the transplantability of murine immature HSC, after isolation from the E9.5 P-Sp and E11.5 AGM and culture for 5 days with AGM-derived endothelial cells and found peritoneal B-1a and B-2 lymphoid cells in the transplanted recipient mice. This led these researchers to propose that the E9.5 immature HSC might generate developmentally restricted fetal liver HSCs, while the E11.5 immature HSCs might develop into conventional fetal liver LT HSCs [99]. Subsequent polylox barcoding and lineage tracing experiments suggested that B-1a lymphoid cells and LT HSCs might originate after E9.5 from a common endothelial precursor [100]. Ghosn et al. [87] have suggested from these and other studies [95,96] that HSC independent or dependent B-1a cells are produced at multiple locations (e.g., yolk sac, P-Sp/AGM, fetal liver) during embryonic and fetal development, with only small numbers of B-1a cells produced from murine adult LT HSCs.

Although these studies support the concept of a layered immune and hematopoietic system, divergent lineage trajectories during different waves of murine hematopoiesis have added increased complexity that is not yet fully resolved.

## 3. Do Human Embryonic and Fetal Hematopoietic Waves Resemble Those in the Mouse?

Although studies on human hematopoietic ontogeny are much more limited than in murine models, human hematopoiesis is reported to commence at approximately Carnegie Stage (CS) 7–8 of embryonic development (16–18.5 days post-conception (dpc)) in the secondary extra-embryonic yolk sac and in close association with yolk sac endothelial cells, termed the yolk sac blood islands [1,2,3,101,102,103]. Based on murine studies, it is generally assumed, although not proven, that human yolk sac-derived hematopoiesis occurs in two waves. The first, or primitive, hematopoietic wave is proposed to generate nucleated erythroid cells, megakaryocytes and macrophages, with the second or pro-definitive hematopoietic wave at around CS13–15 (27–35 dpc) [1,2,3,101,102] generating erythro-myeloid progenitors (EMPs) and potentially certain innate immune or lymphoid-lineage cells from yolk sac hemogenic endothelium (HE) [1,2,3,101,102,103,104,105,106,107].

Definitive human hematopoiesis arises around CS13 (27 dpc) from HE in the AGM (aorta-gonad-mesonephros) region of the embryo proper, for example from the ventral wall of the dorsal aorta, with the generation of HPCs and then importantly the appearance of immature HSCs [101,102,108,109,110,111,112]. From around late CS10 (22 dpc), yolk sac-derived primitive nucleated erythroid cells and CD45^+^ macrophages become evident in the fetal liver rudiment, which is then seeded by CD34^+^CD45^+^ cells from CS13, and finally by definitive AGM-derived HSCs between CS13 and CS17 (27–42 dpc) [101,102,103,104,105,106,107,108,109,110,111,112,113,114]. Here, the cells expand and differentiate, and, from 6 to 7 post-conceptual weeks (pcw) until the middle of the second trimester, the human fetal liver represents the major hematopoietic organ [114]. Fetal liver HSCs colonize the developing fetal bone cavities and have been found at 10–12 pcw at least in fetal long bones, with bone marrow becoming the predominant site for hematopoiesis after 20 pcw and post-natally [1,2,30,112,113,114,115,116,117,118,119,120]. It is unclear if HSC and multipotent progenitor (MPP) subsets are generated from human fetal or young adult bone marrow HE. In adult human bone marrow, however, aging is associated with further changes in hematopoietic outputs and functions [3,4].

## 4. Human Hematopoietic Malignancies during Development and Aging

Different hematological malignancies are more prevalent in different age groups, with some subtypes originating in utero, and with pediatric hematological malignancies generally differing from those in the adult in terms of their etiology and molecular characteristics [121,122]. This has led to a quest to identify the cell of origin of these hematological malignancies, and this is being resolved in part by our improving knowledge of hematopoiesis during human embryonic, fetal and early post-natal development.

**Figure 1:**
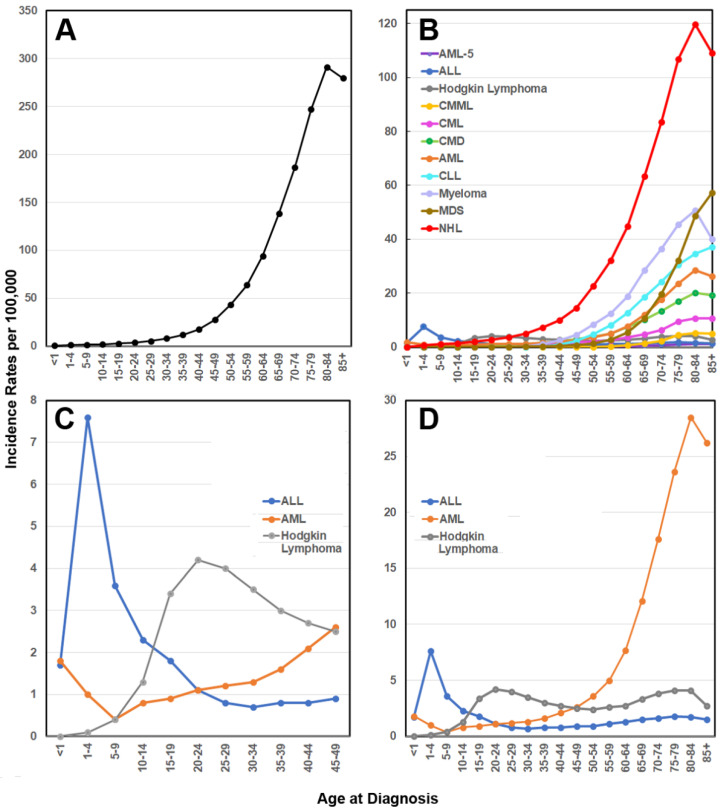
Incidence rates for hematological malignancies by age at diagnosis in the USA from 2014 to 2018 (https://seer.cancer.gov; accessed 28 November 2021) expressed per 100,000 individuals per annum. (**A**) Total cases. (**B**) Cases in A broken down into hematological malignancy subtypes. (**C**,**D**) Incidence of ALL, AML and Hodgkin lymphoma from birth to age 49 and 85+ respectively.

Incidence rates for hematological malignancies by age at diagnosis in the United States of America, from 2014 to 2018, are illustrated in Figure 1. These are based on online data published by the *Surveillance, Epidemiology, and End Results (SEER) Program of the National Cancer Institute and the American Cancer Society* (https://seer.cancer.gov; accessed 28 November 2021), and they update those described by Marcotte and colleagues [121]. As indicated in Figure 1A, most hematological malignancies increase in incidence with aging, with the incidence per 100,000 individuals per annum peaking from around the age of 80, most notably for non-Hodgkin lymphoma (NHL), myeloid dysplastic syndromes (MDS), myeloma, chronic lymphocytic leukemia (CLL), acute myeloid leukemia (AML) and chronic myeloid leukemia (CML) (Figure 1A,B).

In contrast, acute leukemia is the most common pediatric malignancy, with both the *SEER (2014–2018)* (https://seer.cancer.gov) and the *International Incidence of Childhood Cancer-3 (IICC-3)* (2001–2010) studies reporting that the leading hematological cancers in infants and children aged < 10 years of age are leukemias, particularly acute lymphoblastic leukemia (ALL), although in adolescents (the 10–19 year age group; https://www.who.int; accessed 28 November 2021) and young adults, lymphomas are more prevalent [123].

As shown in Figure 1C,D, the pediatric incidence of AML is highest in infants less than 1 year of age, while the childhood incidence of ALL peaks at 1–4 years of age, with these AML and ALL incidences occurring at rates in the SEER analysis for 2014–2018 (https://seer.cancer.gov) of 1.8 and 7.5 cases per 100,000 individuals per annum, respectively. Data from the MRC-UKALL clinical trials also show that a peak incidence of ALL, predominantly B cell precursor ALL, occurs at 2 to 5 years of age [6,124]. However, the global burden of hematological malignancies in children and adolescents/young adults (<20 years of age) is difficult to assess, particularly in low to middle income countries [125].

## 5. Molecular Characteristics of Infant and Childhood B ALL

**Figure 2:**
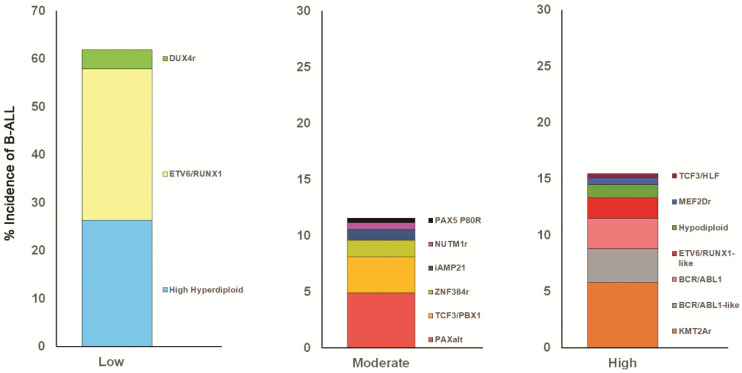
Relative (approximate %) incidence of subtypes (identified in order from top to bottom of each graph by the relevant gene fusions, gene rearrangements or aneuploidy) of pediatric B-ALL data stratified into low, moderate and high risk categories,.and adapted from the St. Jude Total Therapy Study XVI data described by Inaba and Pui [130].

Transcriptomic and next-generation sequencing technologies, together with cytogenetic analyses, have contributed to the 2016 and current updates in progress of the World Health Organization classification of hematological malignancies and serve to highlight the heterogeneity of pediatric acute leukemias [126,127,128,129,130]. High throughput sequencing assays have also been used to identify clinically relevant and novel fusion genes and mutations that are not detected by conventional cytogenetics [128,129,131,132,133,134,135]. More than 30 ALL subtypes have now been identified, with two-thirds being B ALL [130]. Examples of these, stratified into low, intermediate and high risk B ALL categories, are exemplified in Figure 2 based on data sourced from the St. Jude Total Therapy Study XVI data [130], although, with further studies, the risk stratifications within such categories may become more complex [136].

Around 75–80% of pediatric ALL cases are B cell precursor ALL [119,124,135]. Analyses of the more common subtypes by Marcotte et al. [121], based on other earlier studies [137], indicate that approximately 70% of B cell precursor ALLs that occur between ages 1 to 4 years and a slightly lower percentage of those occurring in the 5 to 9 year age group are characterized by high hyperdiploidy and by translocation t(12;21)/*ETV6/RUNX1* fusion gene. A similar proportion (70–80%) of infant B ALLs (<1 years of age) carry *KMT2A* (*MLL*) gene (encoding lysine methyltransferase 2A and located on chromosome 11q23) rearrangements with at least 94 partner genes identified [138,139,140], the most frequent being the translocation t(4;11)/*KMT2A/AFF1* (*MLL/AF4*) fusion gene. Smaller incidences of high hyperdiploidy and t(1;19)/*TCF3/PBX1* gene fusions are also observed [121,130,138,139]. Results from the studies described above are based on the caveat that variations have been reported to occur in the frequency of cytogenetic/molecular abnormalities in pediatric hematological malignancies in different countries and ethnic groups [121].

## 6. The Origins of Pediatric B ALL

Because of its prevalence compared to T ALL and AML, there has been a particular interest in understanding the origin and development of pediatric B cell precursor ALL. Risk factors have recently been reviewed in detail elsewhere [133,141,142,143]. The two-hit model for the development of childhood B cell precursor ALL proposes that an initiating preleukemic event or first hit (e.g., high hyperdiploidy or *ETV6/RUNX1* gene fusion) occurs in utero [6,115]. This concept is supported by studies on monozygotic twins, as well as by backtracking analyses of umbilical cord blood (UCB) and dried neonatal blood spots (Guthrie cards) [6,124]. Other prenatal translocations/rearrangements reported in ALL subtypes include, although not exclusively, *BCR/ABL1* and *TCF3/PBX1* gene fusions and *KMT2A* rearrangements, including the t(4;11)/*KMT2A/AFF1* fusion gene [138,143]. Similar “preleukemic” changes have been detected at birth in the blood of healthy children, who do not subsequently develop ALL [6,121,122,124,141,142,143,144,145,146,147,148]. Notably, about 1% to 5% of newborns are reported to carry *ETV6-RUNX1* gene fusions in approximately 1 in 10,000 B lymphoid lineage cells (although this varies considerably amongst different studies) without overt B cell precursor ALL developing in the vast majority of these children, and with predisposing factors for development of B cell precursor ALL post-natally, including environmental factors and additional mutations [6,121,122,124,141,142,143,144,145,146,147,148]. The second hit is accompanied by independent mutations and subclonal evolution leading to B cell precursor ALL occurring post-natally (at least for the <14-year age group) [6,121,124,143].

For childhood B cell precursor ALL, various hypotheses have been proposed as causal factors in disease progression after birth, including population mixing, infection and delayed infection [149,150,151,152]. In the delayed infection hypothesis, the acquisition of additional mutations, such as those that alter RAG-mediated copy number of cell cycle or B lymphoid lineage differentiation genes [153,154], are proposed to be driven by infections by common pathogens rather than by any specific pathogen [140,141,142,143,144,145,146,147,148,149,150,151,152,153,154,155]. These include upper respiratory and gastrointestinal infections (viral, bacterial, fungal, and potentially including SARS-CoV2 and related viruses), which induce an abnormal immune response or chronic inflammation [124,141,143,155]. These responses to pathogens are predicated on a lack of appropriate infant microbial exposure that possibly affects the establishment of the normal gut and oral microbiome and dysregulates immune cell maturation or triggers B cell precursor ALL in genetically predisposed individuals or those bearing preleukemic translocations [124,142,155]. These and other studies on microbiome modifications in the genesis of ALL and occurring at ALL diagnosis, and during chemotherapy, antibiotic treatments and hematopoietic cell transplantation are described in detail elsewhere [142].

In infants with B ALL (<1 year of age), initial *KMT2A* rearrangements (particularly *KMT2A/AFF1* gene fusions) in utero appear to be sufficient for the onset of leukemia before or shortly after birth [138,139], although secondary mutations are present in some cases [144]. Overall, next generation sequencing studies have revealed a low incidence of somatic mutations in ALL infants with *KMT2A* rearrangements [156,157], although these include mutations in tyrosine kinase PI3K-RAS signaling pathways and to a lesser extent in *FLT3*, as well as abnormal DNA methylation patterns [138,139,158,159,160,161]. Whether preleukemic clones expressing *KMT2A* rearrangements in utero always develop overt infant ALL remains to be fully determined.

## 7. Progress in Defining Human Fetal B Lymphoid Development and the Cell of Origin of Pediatric ALLs Using Single-Cell Omics Approaches

More recently, there has been a surge of interest in using increasingly advanced omics technologies to define more clearly human hematopoiesis and hematopoietic lineage trajectories during embryonic and fetal development. As the initiating events leading to infant and childhood B ALL are thought to mostly originate in utero, the sequence of human B lymphoid development during human embryonic, fetal and early post-natal life has generated intense interest in the quest to define the cell of origin of these childhood leukemias. Progress in defining human fetal B cell development has recently been reviewed [119], and this section of the review highlights, but is limited principally to, more recent studies based on single-cell multi-omics studies.

Based on the detection of phenotypically defined HSC, MPP and lymphoid primed multipotent progenitors (LMPP), and of oligopotent fetal-specific early lymphoid progenitor (ELP) cells [114,162,163,164], the studies of O’Byrne et al. [30] confirmed that lymphopoiesis was evident in human fetal liver at around 6 pcw, with B lymphoid primed cells being detected in fetal liver around 7 pcw, and in fetal blood and bone marrow by 12 pcw [119,165]. Although previous studies had identified CD34^+^CD10^+^CD19^+^ Pro B-progenitors in adult human bone marrow and CD34^+^CD10^-^CD19^+^ PrePro B-progenitors in UCB and second trimester human liver [166,167,168,169,170], O’Byrne et al. [30] comprehensively analyzed the human fetal B cell developmental hierarchy using single-cell RNA sequencing (scRNA-seq) and ATAC-seq to assess transcriptomic profiles and chromatin accessibility profiles respectively, as well as functional assays. Importantly, these studies demonstrated that CD19^+^CD10^−^CD34^+^ PrePro B-progenitors were the earliest B-lymphoid restricted progenitors (lacking myeloid, NK and T cell potential) detected and were positioned upstream of CD19^+^CD10^+^CD34^+^ Pro B-progenitors and downstream from fetal specific Lin^-^CD34^+^CD127^++^/IL-7Rα^++^CD10^–^CD19^–^ ELPs with the ability to give rise to B, T, NK and some myeloid cells. O’Byrne et al. [30] used matched human fetal liver and fetal bone marrow samples to demonstrate that the emergence of fetal liver PrePro B-progenitors was followed from 11 pcw by their presence and then their proliferation in fetal bone marrow, with PreProB and ProB progenitors eventually comprising more than 30% of fetal bone marrow CD34^+^ cells in the latter part of the second trimester. While complete V_H_-D_H_-J_H_ rearrangements occur in Pro B-progenitors, only partial D_H_-J_H_ IgH rearrangements occur in fetal ELP and PrePro B-progenitors [30]. Notably, HSC, MPP and LMPP were also detected in both fetal liver and fetal bone marrow, where they were capable of producing B, T, NK and myeloid cells [30]. These studies, while adding significantly to defining human fetal B lymphopoiesis, did not exclude the existence of human yolk sac lymphoid progenitors, which have been suggested from other transcriptomic studies [114,119].

Other studies have examined and set out to better characterize human B-1 and B-2 lymphoid development. As indicated earlier, innate-like B-1 (B-1a and B-1b) and conventional B-2 cells constitute two main branches of the murine B cell population. During embryonic and fetal development, murine B-1a subsets are proposed to arise from multiple sites including yolk sac, P-Sp/AGM and fetal liver from HSC independent or dependent precursors, and to a lesser degree from adult bone marrow HSCs [85,87]. Although much less is known about the equivalent human B-1 B cells, these cells were provisionally identified as CD20^+^CD27^+^CD43^+^CD70^–^ cells in UCB and adult peripheral blood [171], and in other studies, in fetal liver and fetal bone marrow as well as UCB, reaching their highest frequency in fetal liver around 10 pcw [172]. As this phenotypic cell subset may also contain CD20^+^CD38^hi^ plasmablast and preplasmablast precursors, it remains possible that the human fetal B-1 B cell frequency has been over estimated [173,174,175,176,177,178]. Putative human B-1 cells, the earliest B cells to arise in the human fetus, were therefore subsequently identified phenotypically as CD19^+^CD20^+^CD27^+^CD43^+^CD38^lo/int^, while lacking CD3, CD4 and CD8 T cell markers, making them distinct from B-2 lymphoid cells, which are predominantly found in adult tissues [173,174,175,176,177,178]. Whether human B-1 and B-2 B cells arise from different progenitors or a common progenitor is unclear. However, recent evidence examining hematopoietic lineage output at the clonal level from the peripheral blood of adult patients with paroxysmal nocturnal hemoglobinuria (PNH) suggests that this putative human B-1 B cell population can also arise in the adult from HSCs [178], while other studies indicate that human adult B-1 B cell frequency and diversity decline with aging, particularly after the age of 50 [172].

There have been suggestions that at least a proportion of infant and childhood B ALLs arise from B-1 B cells or their precursors [179]. Given this, Fitch et al. [180] compared whole transcriptomic profiles among different human pediatric B ALL subtypes with murine B-1 and B-2 progenitor signature genes, comprising a set of 30 differentially expressed genes. From their comparative transcriptomic profile analyses, they suggest that human pediatric *ETV6/RUNX, TCF3/PBX1, CRLF2* and *ERG* B ALLs are more likely to originate from B-1-like cells, while *BCR/ABL1*, hyperdiploid, and *KTM2A* rearranged B ALLs derive from B-2-like B cells [180,181]. These researchers suggest that specific B ALL subtypes may either arise in human B-1 or B-2 B cells following an initiating translocation or mutation, or that the specific translocation or mutation activates B-1 or B-2 B cell transcriptional programs in an appropriate B progenitor cell during fetal development [180,181]. Two of the translocations examined by Fitch et al. [180] are of special interest for further study as they more frequently occur in infant B ALL (*KMT2A/AFF1*) and childhood B cell precursor ALL (*ETV6/RUNX1*) yet are predicted from the studies above to arise in different B lymphoid lineages, the former in conventional B 2-like and the latter in innate B 1-like B cell lineages.

In relation to *KMT2A/AFF1* infant B ALL, fetal PrePro B-progenitors express several genes implicated in infant ALL, while *KMT2A*-rearranged B ALL clones, similar to PrePro B progenitors, are CD10- and carry partial D_H_-J_H_ rearrangements [30]. These observations led to the suggestion that fetal PrePro B-progenitors or their precursors may constitute the cell of origin for initiation of *KMT2A* rearranged infant B ALL [30]. Considering this, and that both *KMT2A* rearranged infant and childhood ALLs originate in utero (albeit with the former having a much more aggressive disease course) [182]. Rice et al. [183] hypothesized that *KMT2A* (*MLL*)-rearranged infant ALL is initiated and maintained by co-operation between fetal specific gene expression programs or environment and the rearranged *KMT2A* gene. Using *KMT2A/AFF1* (*MLL/AF4*), the most common infant ALL rearrangement as a model, their studies demonstrated that *KMT2A/AFF1* infant ALL did maintain a designated fetal specific gene expression profile, while *KMT2A/AFF1* childhood ALL did not [182,183]. Furthermore, primary human fetal liver CD34+ HSPCs were gene edited using CRISPR-Cas9 to produce HSPCs carrying a t(4;11)/*KMT2A/AFF1* translocation; they were shown to drive fetal specific and infant ALL molecular programs and to recapitulate clinical characteristics of the human disease (including treatment resistance and CNS disease) in an NSG xenograft model of infant ALL [183], providing support for their hypothesis. While the studies described above provide mechanistic insights and an important advance in modelling infant ALL, because of the heterogeneity of the fetal liver CD34+ HSPC cells, no conclusions can yet be drawn regarding the exact cell of origin of these infant ALLs.

Other studies, which have included an analysis of human fetal bone marrow (13–29 pcw) ELP subsets, have proposed a “two-family” model for lymphopoiesis, in which CD127^-^ and CD127^+^ (IL-7Rα^+^) ELPs generate human lymphoid cells, with both CD127 subsets arising independently from multipotent CD34^hi^CD45RA^+^ Lympho-Mono-Dendritic cell progenitors (LMDPs) [183]. While CD127^+^ ELPs generated NK, ILC and B cells, but not T cell subsets, CD127^-^ ELPs gave rise to T cells, ILCs, and NK and marginal zone B cells [184]. Except for their lack of T cell potential [184], the former CD127^+^ ELPs closely resemble the fetal bone marrow ELPs that are putative PrePro B cell precursors [30], perhaps suggesting that additional heterogeneity exists in the fetal ELP subpopulation. More recent transcriptomic studies [185] comparing *KMT2A* rearranged infant B ALL with previously published fetal human bone marrow transcriptomes [120] have concluded that *KMT2A* rearranged infant B ALL resembles a fetal ELP state as defined above [30,184]. Given that there are at least two putative human fetal ELP subsets based on CD127 expression, it remains to be determined if both fetal ELP subsets are affected by the *KMT2A/AFF1* fusion gene, or whether the leukemic/preleukemic event occurs in another progenitor type that then arrests at the ELP stage, and, if so, if this has any bearing on the development of infant versus childhood ALL. In this respect, assessment of the phylogenetic origin of a rare case of lineage switching from *KMT2A* rearranged infant B ALL to *KMT2A* rearranged childhood AML has led these same researchers to suggest that, in this specific case, the *KMT2A* rearrangement may have occurred before gastrulation and hematopoietic specification [185].

In other studies involving *ETV6/RUNX1* B cell precursor ALL, Boiers and colleagues [186] identified human fetal liver CD19^-^IL-7R^+^/CD127^+^ (Lin^-^CD19^-^CD34^+^CD38^+^CD45RA^+^IL-7R^+^KIT^+^) cells, which during ontogeny (CS17 to CS20), transition from a myeloid-primed to a lymphoid-primed program, as the earliest human B lymphoid progenitors. Additionally, a proportion of CD19^-^IL-7R^+^/CD127^+^ progenitor showed evidence of D_H_-J_H_ rearrangements and myeloid (principally macrophage) cells in vitro [186]. Transcriptionally and functionally similar CD19^-^IL-7R^+^ progenitors were also identified when human iPS cells were differentiated in an OP9/MS5 co-culture system, with day 10 differentiated human iPS cells resembling CS17 fetal liver CD19^-^IL-7R^+^ progenitors and day 31 differentiated human iPS cells resembling CS17 and CS20 fetal liver CD19-IL-7R^+^ progenitors (mixed myeloid and lymphomyeloid primed) [186,187]. Subsequent expression of an *ETV6/RUNX1* fusion gene at physiological levels in, and differentiation of, these human iPS cells led to an expansion of the CD19^-^IL-7R^+^ progenitor cells, a partial B lymphoid lineage commitment block, and the generation of proB cells aberrantly co-expressing myeloid gene signatures and potential, and thus potentially recapitulating the *ETV6/RUNX1* preleukemic state [186]. The relationship of these CD19^-^IL-7R^+^ human B lymphoid progenitors to B-1 and B-2 B cells is yet to be established, although it has been reported that fetal liver and adult human pro B cells that express both CD27 (IL-7Rα) and LIN28B preferentially mature to a B1-like B cells [188].

Thus, these studies, while incomplete and still ongoing, have substantially increased our understanding of human fetal B lymphoid development and progenitor cells, and the development of pediatric B ALL.

## 8. Pediatric AML and Juvenile Myelomonocytic Leukemia (JMML)

Myeloid leukemias occurring neonatally or in early childhood include infant AML [127], juvenile myelomonocytic leukemia (JMML), which generally results from activating mutations in *Ras* signaling pathways [189,190], and myeloid leukemia of Down Syndrome (ML-DS), which is reviewed in detail elsewhere and not discussed further here [191]. As well as genetic conditions such as Noonan syndrome, risk factors for pediatric myeloid leukemia include exposure in utero to ionizing radiation [5,141]. Since evidence indicates that all or some of the myeloid leukemias listed above are initiated prenatally and because their heterogeneity has made their study difficult, we highlight some of the more recent studies that are increasing our understanding of the origins and progression of these leukemias.

Over 20 different subtypes of AML have been defined based on the 2016 revision of the WHO classification of myeloid neoplasms and acute leukemias [126], with approximately 20% of pediatric leukemias being AMLs [121]. More than 50% of infant, childhood and adolescent patients (<18 years old) with AML have abnormal karyotypes, which include aneuploidy (monosomy 5 and 7, trisomy 8 and 21) and such chromosomal rearrangements as t(9;11)/*KMT2A*/*MLLT3*, t(15;17)/*PML/RARA*, t(8;21)/*RUNX1/RUNX1T1*, and inv(16)/t(16;16)/*CBFB/MYH11* (see Marcotte et al. [121] and other reports [127,192,193]). Notably in these studies, almost half of infant AML cases (0–1 years old) carry *KMT2A* rearrangements, with a significant proportion bearing the t(9;11)/*KMT2A/MLLT3* (*MLL/AF9*) fusion gene, the incidence of which declines during childhood and adolescence [121]. Of further note, is an increased incidence in these latter age groups of t(15;17)/*PML/RARA*, t(8;21)/*RUNX1/RUNX1T1*, inv(16)/t(16;16)/*CBFB/MYH11*, and trisomy 8 karyotypic subtypes [121]. Both complex karyotypes and normal karyotypes with defined mutations have also been described in pediatric AML [121,127]. Gene alterations with a higher prevalence in AML in these age groups include new mutations in *GATA2, FLT3* and *CBL,* and recurrent mutations in *KRAS, NRAS, KIT, WT1* and *MYC-ITD* [127,133]. Lineage switching of leukemias may also occur; for example, leukemia associated with the *KMT2A/AFF1* fusion gene, which as described above, commonly presents as pediatric B cell precursor ALL, may also demonstrate an infant B/myeloid mixed phenotype or relapse with the original clone switching to AML [139,140,194,195,196,197,198,199].

Although significantly fewer than for pediatric ALL, backtracking studies have detected t(15;17)/*PML/RARA* and t(8;21)/*RUNX1/RUNX1T1* fusion genes in neonatal blood spots or UCB of children or adolescents subsequently developing AML, indicating that these as a minimum can be initiated in utero [121,200,201,202,203]. Further backtracking research on the prenatal origin of pediatric AML, using neonatal blood spots and UCBs sourced at birth, is ongoing within registered clinical trials (https://clinicaltrials.gov/ct2/show/NCT05014165; accessed 28 November 2021). In utero, preleukemic events would of course also include such genetic or inherited predispositions as Noonan and CBL Syndromes and Neurofibromatosis type-1 (NF-1), which place such children at higher risk of developing, although not necessarily exclusively, myeloid leukemias [121,133,204,205]. Individuals with NF-1 or CBL and Noonan Syndromes, which dysregulate the *RAS* pathway genes, are predisposed to JMML [204,205]. While the majority of those with Noonan Syndrome carry germline mutations in *RAS* pathway genes (e.g., *PTPN11, KRAS, NRAS, SOS-1, RAF1, BRAF*), not all develop JMML. About 5% develop a transient myeloproliferative disorder, which spontaneously resolves, although in some cases, this will progress to JMML [204,205]. This contrasts with children without Noonan Syndrome bearing somatic *RAS* mutations in *PTPN11, NRAS* and *KRAS*, who account for a significant proportion of JMML cases [205]. An analysis of neonatal blood spots has identified such somatic *RAS* pathway mutations (most commonly in *PTPN11, NRAS* and *KRAS*) in 38% of children (*n* = 34) without Noonan Syndrome but presenting with JMML at a median age of 1.5 years [206]. These children were significantly younger at the time of JMML diagnosis than those for whom the somatic mutations were not detected at birth [206]. This suggests that a significant number of these children had developed preleukemic changes prenatally and these more rapidly progressed to JMML.

Given its importance in infant AML, murine and human model systems, combined with single-cell omic assays [207,208,209], have been developed to examine the effects of the *KMT2A/MLLT3* fusion gene on AML development [210]. These include the recent development of a transplantable human *KMT2A/MLLT3* AML xenograft model, using human UCB CD34^+^ HSPCs and CRISPR/Cas9 genome editing technologies, which also reported that the developmental age and the genetic background of the human CD34+ HSPCs, as well as the microenvironmental niche in surrogate murine models of hematopoiesis influenced AML progression [211]. Similarly, while Wei and colleagues demonstrated the exclusive generation of AML by transducing human UCB CD34+ HSPCs with the *KMT2A/MLLT3* fusion gene prior to transplantation into NS-SGM3 [212], Horton et al. [213] showed that human UCB CD34+ HSPCs transduced with the *KMT2A/MLLT3* fusion gene generated both AML and ALL in NSG mice, whereas similarly transduced adult bone marrow HSPCs generated LT hematopoietic engraftment with a myeloid bias, which did not progress to AML. More recently, using a retroviral based *KMT2A/MLLT3* model derived from human UCB CD34+ HSPCs, in which the genetic background of the UCB donor was defined by next generation sequencing prior to the introduction of the *KMT2A/MLLT3* fusion gene, Milan et al. [214] also concluded that HSPCs “primed” by the *KMT2A/MLLT3* fusion gene require additional signals, possibly from the bone marrow niche, for leukemic transformation. Consistent with this, Hyrenius-Wittsten et al. [215] demonstrated that co-expression of *KMT2A/MLLT3* and *FLT3^N676K^* in human CD34+ UCB HSPCs mainly resulted in the development of AML in NSG mice, concluding that constitutively active signaling mutations within the transduced cell could replace exogenous factors and promote AML.

Importantly, significant progress is being made in understanding the molecular basis for the initiation and progression of pediatric *KMT2A/MLLT3* AML, as well as defining the individual functions of the *KMT2A* and *MLLT3* in normal human hematopoiesis. As an example, MLLT3 is a key regulator of human HSC self-renewal and engraftment, potentially acting as an HSC maintenance factor by protecting the stemness program as HSC divide, and a critical regulator of early human erythroid and megakaryocyte fate [216,217,218]. Other research has demonstrated that KMT2A (lysine methyltransferase 2A) forms part of a complex that regulates *HOX* gene transcriptional activation, while the KMT2A/MLLT3 fusion protein forms part of the disruptor of telomere silencing 1-like (DOT1L, a histone 3 lysine 79 methyltransferase) complex (DOTCOM), the effects of which are reviewed elsewhere [219,220,221]. Given that *KMT2A/MLLT3* AML may be initiated in utero [202], it would be of interest to define the human fetal or embryonic cell of origin in which the *KMT2A/MLLT3* preleukemic event occurs and determine if additional mutations and/or the fetal or early neonatal hematopoietic microenvironment differentially influence progression to AML in infants as opposed to older children. Such an approach has recently been taken by one of us in the context of infant *KMT2A/AFF1*-driven ALL and AML [183,222,223].

JMML, which accounts for 1% of pediatric leukemias, has a median age of onset of 2 years [189,204,205,224,225]. Over 90% of JMML driver mutations involve five genes in the canonical *RAS* pathway (*PTPN11, NRAS, KRAS, NF1*, *CBL)*, with approximately 35% being somatic *PTPN11* (gain of SHP-2 function) exon 3 or 13 mutations [133,189,190,204,205,224,225,226,227,228,229]. Hypersensitivity of JMML progenitors to GM-CSF, IL3 and TNFa in vitro, hyperproliferation of monocytic and/or granulocytic lineages in vivo, thrombocytopenia, and increased fetal hemoglobin (HbF in 50–60% of patients) are common JMML features, with occasional transformation to ALL, suggesting a disease of, or expressed in, multipotent HSC/MPP [204,205,224,225,226,227,228,229]. A number of recent studies have investigated the cellular origin and clonal evolution of JMML using iPS cell [230,231,232,233,234,235,236] and xenograft models [237,238,239]. Caye et al. [238] demonstrated the propagation of transplanted primary bone marrow JMML HSPCs (particularly those in the *PTPN11, NRAS* and *KRAS* subgroups and with a median age of 2.2 years) in immunodeficient NSG and NSG-SGM3 mice. This was further investigated by Louka et al. [239], who transplanted enriched defined JMML peripheral blood or bone marrow subsets (Lin^−^CD34^+^CD38^−^CD90^+^CD45RA^−^ HSC, Lin^−^CD34^+^CD38^+^CD123^+^ CD45RA^+^ GMP and as a novel JMML specific double-positive Lin^−^CD34^+^CD38^−^CD90^+^CD45RA^+^ myeloid progenitor subset) into NSG mice, demonstrating that each HSPC subset propagated JMML in vivo. Single-cell index sorting of Lin^−^CD34^+^ HSPCs and colony genotyping revealed that the JMML HSPC compartment was clonally heterogeneous, containing both clonally dominant *RAS* pathway mutations and subclones bearing other mutations (*ASXL1*, *SETBP1*, and monosomy 7), with all somatic mutations being backtracked to the phenotypically defined HSC subset [239]. Acquisition of mutations in addition to the *RAS* pathway mutations followed both linear and branching patterns of clonal evolution [239]. These studies demonstrate that aberrant HSPC subsets (HSC/MPP, myeloid progenitors) with a myeloid bias propagate JMML [238,239], but do not definitively identify the JMML cell of origin. Notably, however, higher expression of fetal HSC genes, *HMGA2, CNN3* and *VNN2*, and overexpression of *HOPX*, which encodes a non-DNA binding homeodomain protein involved in primitive hematopoiesis has been demonstrated in JMML HSCs, supporting a putative JMML embryonic or fetal origin [116,239,240,241]. Additionally, a stemness gene signature (*HOPX, SPINX2, CLERC9A*) was present in both JMML/HSC and JMML myeloid progenitors, with gene regulatory networks/regulons (*FLI1, MEF2C, MECOM,* and *GATA2*) in these JMML subsets being reminiscent of fetal HSC/MPP [116].

## 9. Shifting Human HSC Heterogeneity with Aging and Clonal Hematopoiesis of Indeterminate Potential

As described above, sequential waves of human HPCs and HSCs are generated from such tissues as the yolk sac and the AGM, coupled with their expansion in the fetal liver in order to meet the needs of the developing embryo and fetus. The shift of this hematopoiesis from the fetal liver to the fetal bone marrow from 10–12 pcw represents the first step toward establishing the bone marrow as the predominant site for adult human hematopoiesis. Recently, single-cell multi-omics of human fetal liver and fetal, pediatric and adult bone marrow have highlighted the significant changes in the composition and function of HSPCs from fetal to adult life [115,116]. Notably, as hematopoiesis relocates from the human fetal liver to the fetal bone marrow, HSC/MPPs shift from a highly proliferative to a quiescent state [115,116], and their lineage output shifts principally from erythroid-megakaryocytic to lympho-myeloid lineages [114,120]. A rapid and extensive diversification of myeloid cells occurs with the first appearance of granulocytes, eosinophils and dendritic cell subpopulations (plasmacytoid, transitional and DC3) in human fetal bone marrow [120]. A 10-fold higher frequency of B lymphoid lineage cells is also observed, but with a marked skewing toward earlier differentiation states than seen in adult bone marrow [120]. These early B cell progenitors from human fetal bone marrow were found to highly express small translocations and deletions in a set of genes that cause B ALL in infancy and childhood [120].

At the other end of the spectrum, the aging hematopoietic system of the adult bone marrow not only shows increased susceptibility to certain hematopoietic disorders but is also linked to the development of other diseases that include stroke and cardiovascular diseases [242,243]. The World Health Organization suggests that more than 17% of the global population (about 1.4 billion individuals) will be 60 years or older by 2030 (https://www.who.int; accessed 28 November 2021). The aging process coincides with an increase in HSC numbers and adipogenesis in adult human bone marrow; this is accompanied by a loss of clonal HSC functional heterogeneity, decreased regenerative capacity and a reduction in lymphopoiesis, characterized by a shift from lymphopoiesis to myelopoiesis [4,41,244,245,246,247,248,249,250]. With respect to hematopoietic diseases, the consequences of aging may include the development of immune and autoimmune disorders, clonal hematopoiesis of indeterminate potential (CHIP), acute and chronic leukemias, multiple myeloma, non-Hodgkin lymphomas (NHL), myeloproliferative neoplasms (MPN), and myelodysplastic syndrome (MDS), thus differing substantially from the hematological malignancies of infancy and childhood (Figure 1) [3,4,121,251].

Somatic mutations are prevalent in the highly proliferative hematopoietic system and accumulate during development (as indicated earlier) and, to a greater extent, with aging [251,252,253]. For many years, there has been substantial interest in the developmental origin of gene translocations and mutations that predispose to pediatric hematological malignancies, but this is only now being realized with human adult hematopoietic neoplasms. For example, whole genome sequencing of single hematopoietic colonies from MPN patients has revealed a considerable latency period from the acquisition of the driver somatic mutation until disease development [254,255]. In such patients, driver mutations in *DNMT3A, PPM1D* and *JAK2^V617F^* originated either in utero or in childhood [254]. Although the number of patients was small, the estimated acquisition of *DNMT3A* mutations ranged from 8 pcw to 7.6 years of age, and of the *JAK2^V617F^* mutations from at least 33 pcw to 10.8 years of age, while a *PPM1D* mutation was estimated to occur at 5.8 years of age [254]. In the case of *JAK2^V617F^*, the MPNs were diagnosed 11 to 54 years later [254]. Lineage histories reconstructed from individual HSCs by Van Egeren et al. [255] additionally demonstrated that the *JAK2^V617F^* mutation occurred decades before MPN diagnosis (at age 9 years in a 34 year old patient, and at age 19 in a 63 year old patient), with the HSC carrying the mutation having a selective fitness or growth advantage.

In adults, HSPC-associated somatic mutations can lead to clonal hematopoiesis, which increases with age, occurring in more than 10% and possibly as many as 15–20% of individuals aged 70 years or older, and defined as somatic genomic changes in cells of the hematopoietic lineage of individuals with no evidence of hematological malignancy [4,256,257,258]. The prevalence of clonal hematopoiesis in individuals younger than 40 years of age is negligible [256,257,258]. A proportion of those individuals with clonal hematopoiesis is diagnosed with CHIP, which is defined as “a clonal population of blood cells bearing a point mutation or short insertion/deletion with a variant allele fraction (VAF) ≥2% in a gene that is recurrently mutated in hematologic malignancies” [259]. While individuals with CHIP have been considered at risk of developing myeloid leukemias [242,243,260], lymphoid clonal hematopoiesis has been associated with mosaic chromosomal alterations (mCAs), leading to an increased risk of lymphoid malignancies [261,262]. While these and other genetic alterations associated with clonal hematopoiesis have been reviewed recently [251], one recent advance of note (building on earlier studies [256,257,258,260]) used peripheral blood samples deposited at the United Kingdom Biobank and Mass General Brigham Biobank by individuals (40–70 years old) without a history of hematological malignancy to analyze, by whole exome sequencing and single nucleotide polymorphisms, 55,383 individuals for CHIP and 420,969 individuals for autosomal mCAs [259]. Importantly, by examining selected somatic myeloid and lymphoid driver gene variants, the affected individuals could be divided into those with myeloid (M)-CHIP, with lymphoid (L)-CHIP or with both L- and M-CHIP. Of these, M-CHIP was the most prevalent, with the top three mutated genes being *DNMT3A* (DNA methyltransferase 3 alpha), *TET2* (ten-eleven translocation dioxygenase 2) and *ASXL1* (additional sex combs-like transcriptional regulator 1) [259]. Each of these genes is involved in the regulation of human HSC self-renewal, with *TET2* loss of function also skewing hematopoietic differentiation toward the myelomonocytic lineage as reviewed recently elsewhere [251]. Similarly, mCAs can be divided into myeloid, lymphoid or mixed mCAs (M-mCA, L-mCA and mixed M- and L-mCAs) [259]. Follow up studies revealed that, within a median time of about 5–7 years, a proportion of those individuals with M-CHIP or M-mCA developed myeloid malignancies, while a subgroup of those with L-CHIP or L-mCA developed lymphoid malignancies, although the annual incidence of both was low [259]. M-CHIP (large clones) and M-mCA were found to be more frequently associated with AML, MDS and MPN, and L-CHIP and L-mCA most frequently associated with chronic lymphocytic leukemia (CLL) and small lymphocytic lymphoma (SLL) [259]. Previous studies have indicated a 10-fold risk of progression to hematological malignancies in individuals with CHIP [256,257]. Additionally, M-CHIP, but not L-CHIP, represented a risk factor for coronary artery disease [259]. Other studies have also reported such a link between CHIP (with characteristics consistent with M-CHIP) and the increased risk of coronary artery disease, chronic heart failure, degenerative aortic valve stenosis, atrial fibrillation or stroke, and of autoimmune diseases and potentially osteoarthritis, and/or have aimed to better define the mechanisms of action, to improve patient management and to identify therapeutic targets that are still in their infancy as reviewed [243,251,263,264,265,266,267,268,269,270,271,272].

Although not the focus of this manuscript, alterations in the aged bone marrow microenvironment (e.g., in stromal and immune cells, cytokine/chemokine/extracellular matrix production and inflamm-aging) may lead to changes (e.g., of location, regulation, function) in HSPCs and support their evolution to hematological malignancies. The studies, which support these conclusions [19,250,273,274,275,276,277], are not reiterated here, except to highlight the controversy that surrounds the clonal origin of MDS MSC and MDS associated gene mutations or chromosomal rearrangements in MSCs that contribute to MDS progression, and which are described in detail in recent reviews [250,278,279]. Initial experiments in mice demonstrated that disruption of hematopoiesis associated with progression to an MDS-like disorder (characterized by anemia, thrombocytopenia, reduced B lymphoid cells and increased myeloid cells) occurred when the *Dicer1* gene was deleted in murine osteoprogenitor cells [280]. Culturing normal HSPCs with MSCs from these mutant mice resulted in altered hematopoietic cell function and morphology, while normal hematopoiesis ensued after the transplantation of bone marrow HSPCs from the mutant mice into wild-type murine recipients [280]. In contrast to these murine studies, recent research on the initiation of MDS in humans was not found to be associated with pathogenic germline DICER1 variants [281,282]. Studies by Balderman et al. [283], using a transgenic murine MDS model, further support the concept of an altered bone marrow microenvironment contributing to myeloid skewing during MDS progression. Thus, perturbed MSC subsets can adversely affect hematopoiesis in murine models. In humans, decreased adult bone marrow osteoblast and osteoclast numbers have been associated with MDS development [284], while a significantly reduced ability of MDS-derived MSCs to support human CD34^+^ HSPC proliferation in long term in vitro cultures has also been reported [285]. More recently, Wobus et al. [286] have demonstrated that adult bone marrow MSCs from human MDS patients treated with luspatercept, a novel recombinant Fc fusion protein containing a modified type IIB activin receptor, increased colony forming cell (CFC) potential in vitro of healthy but not MDS HSPCs, while pretreating MSCs from MDS patients with luspatercept restored the CFC potential of HSPCs in co-culture and increased both their CXCL12 secretion and HSPC homing in a surrogate zebrafish model. Other research has debated whether the acquisition of mutations in human bone marrow stromal cells contributes to MDS progression. Some reports detect acquired somatic mutations in adult bone marrow MSCs sourced from MDS patients [287,288,289], while Jann et al. [290] identified mutations that were a secondary consequence of MDS MSC expansion ex vivo but could provide no evidence to support the acquisition of mutations that initiate MDS; they did however find biological and functional alterations in MSCs from MDS individuals compared to those from healthy donors [290]. A variety of intrinsic changes to the adult bone marrow stromal cell niche that impact hematopoiesis have been reported to occur with aging; these include compromised skeletal stem cell and MSC secretome functions, and the onset of MSC senescence related to dysregulation of epigenetic control mechanisms and metabolic states, and/or to chronic low grade inflammation (inflamm-aging) [250,273,274,275,276,277,279].

## 10. Impact on HSC Function of Changes in Metabolism and Inflammation during Aging

A cell autonomous increase in human adult bone marrow HSC self-renewal has been considered as the main driving force for clonal hematopoiesis, rather than neutral genetic drift [252]. Although clonal hematopoiesis is often mediated by dysregulated epigenetic control mechanisms, other stressors, such as chronic low-grade inflammation (inflamm-aging) and alterations in cellular metabolism during aging, are thought to confer a competitive advantage to the expansion of the affected hematological clones [4,10,291,292,293,294,295,296,297,298,299,300,301]. Given the excellent comprehensive reviews on developmental- and age-related changes in HSC metabolism and/or inflamm-aging [10,292,293,294,295,296,297,298,299], we restrict this section to a brief overview of more recent studies on the role of mitochondria and lysosomes in regulating HSC fate before focusing on the contribution of inflamm-aging and altered metabolism to clonal hematopoiesis driven by mutations in the epigenetic modifier genes, *DNMT3A*, an epigenetic writer, and *TET2*, an epigenetic eraser, both of which, as indicated above, play significant roles in CHIP development.

Metabolism controls HSPC function, in part by providing energy (ATP) and tricarboxylic acid (TCA) cycle metabolites [293,294]. ATP is produced by glycolysis, the conversion of glucose to pyruvate in the cell cytoplasm, and by oxidative phosphorylation, which involves the oxidation of pyruvate to acetyl-CoA via the mitochondrial TCA cycle [293,294,302]. Under homeostatic conditions, adult bone marrow HSCs, which exist in a quiescent state in order to maintain their potency and as a protection from replicative and oxidative stress, principally rely on anaerobic glycolysis for their energy rather than mitochondrial oxidative phosphorylation, consequently limiting their levels of reactive oxygen species (ROS) [293,294,302,303]. In contrast, fatty acid oxidation promotes HSC self-renewal and asymmetrical division [294,304], while HSC differentiation is associated with a switch from glycolysis to mitochondrial oxidative phosphorylation, which moderately increases ROS levels [293,302,305]. Mitochondria and lysosomes, as nutrient signaling and sensing hubs, and crosstalk between these organelles, are now considered important elements for regulating HSC fate and function [10,293,294,302,306,307]. By exploiting scRNA-seq and the heterogeneity of mitochondria (particularly in mitochondrial membrane potential) in murine HSC subsets, Liang and colleagues [307] recently distinguished deeply quiescent HSCs from activated (cycling-primed) HSCs, revealing that cycling-primed HSCs, rather than quiescent HSCs, are glycolysis dependent; HSC quiescence was shown to be maintained by large, inactive and abundant lysosomes, which have the ability to suppress glucose uptake and to sequester mitochondria, characteristics associated with an enhanced long-term in vivo reconstitution ability [307]. This suggests that the survival of cycling-primed HSCs, but not deeply quiescent HSCs, requires glucose consumption and pyruvate transport by mitochondria. Other research shows further co-operation between mitochondria and lysosomes in regulating HSC fate (see detailed reviews [294,302]). Garcia-Prat and colleagues recently demonstrated that lysosomal activity in human HSCs is differentially regulated by the transcription factors, TFEB and MYC. TFEB limits metabolic activation by inducing the degradation of key cell surface membrane receptors by lysosomes and thereby promotes LT HSC quiescence or self-renewal, while MYC represses lysosomal catabolism and drives LT HSC activation and differentiation [308]. Other studies suggest that the asymmetric inheritance of lysosomes and mitochondria by daughter cells may add another layer of complexity to the mechanisms that regulate HSC fate, potentially contributing to HSC aging and the progression to CHIP [294,302,309], although this requires more detailed investigation. In another cellular system, the differential distribution of old versus new mitochondria by the asymmetrical division of murine epithelial stem cells has recently been shown to influence the cell’s decision to differentiate or maintain stemness, and this in part is mediated by an alteration in the levels of metabolites involved in chromatin or epigenetic regulation [310,311].

The role of different classes of epigenetic regulators in hematopoiesis and age-related clonal hematopoiesis has been recently reviewed [312,313,314]. As mentioned above, mutations in the epigenetic modifiers *DNMT3A* and *TET2* are early drivers of age-related clonal hematopoiesis. DNMT3A, one of three mammalian DNA methyltransferases, catalyzes the de novo methylation of DNA principally by converting cytosine residues to 5-methylcytosine; its major methyl donor is S-adenylmethionine, a product of mitochondrial one-carbon metabolism [302,315]. Surrogate murine in vivo transplantation models demonstrate that *Dnmt3a-/-* HSCs exhibit extensive self-renewal ability as well as a reduced ability to differentiate [10,296,316,317,318,319]. Additional studies have revealed that inflammation, mediated by IFNγ signaling and associated with chronic mycobacterial infection, drives the self-renewal and clonal expansion of murine *Dnmt3a-/-* (but not *Dnmt3a+/+*) HSCs and MPPs by promoting widespread changes in global methylation and a reduction in stress-related apoptosis [320,321]. Recent analyses of human cells also show that *DNMT3A* mutations are spread across the gene, with a significant proportion (74%) being loss-of-function mutations, which in 50% of cases, exhibit a reduction in DNMT3A protein stability and increased protein degradation that correlates with enhanced clonal expansion [322].

In contrast to DNMT3A, TET2 dioxygenase demethylates DNA by oxidizing 5-methylcytosine to 5-hydroxy-methylcytosine (see recent reviews [312,323]). Fluxes in substrates and cofactors provided by metabolic pathways or changes to glycolytic enzymes during aging influence TET2 enzymatic reactions [10,296,315,323,324,325], thereby altering DNA methylation patterns and subsequently HSPC fate. TET2 catalytic activity, for example, is dependent on α-ketoglutarate, is activated by Fe^++^ and ascorbate, and is inhibited by 2-hydroxyglutarate, fumarate and succinate, with increases in glycolysis limiting α-ketoglutarate availability and with upregulation of oxidative phosphorylation enhancing α-ketoglutarate levels [293,294,302]. TET2-deficient human or murine HSC clones exhibit enhanced proliferation and a myeloid bias [325], while single *Tet2-/-* murine HSCs demonstrate significant changes to DNA methylation of lineage specific transcription factor binding motifs, with resultant disruption to transcriptional priming [323,324]. *TET2* loss-of-function mutations also increase secretion of proinflammatory cytokines by mutant *TET2* hematopoietic cells, which can alter HSPC fitness and enhance clonal expansion [272]. In this respect, defects in maintenance of the intestinal barrier, which occur when *Tet2* is deleted in hematopoietic cells, allow bacteria to enter the blood stream provoking a microbial-dependent pro-inflammatory response and resulting in further survival and proliferative advantages to the *Tet2-/-* HSPC clones [326,327]. Thus, it seems feasible that chronic infection and inflammation (inflamm-aging) coupled with dysbiosis [244] and metabolic changes related to age-dependent hematopoietic decline co-operate to promote the expansion of *DNMT3A* and *TET2* loss-of-function hematological clones in older individuals.

## 11. Conclusions

In this review, we have attached a special importance to more fully understanding normal human hematopoiesis at each end of the age spectrum, and to highlight evidence for certain premalignant chromosomal rearrangements/translocations or driver mutations arising in utero or in early post-natal life, yet with the malignant hematological disease not manifesting itself clinically for weeks to many decades later. We have further sought to describe the importance of better defining hematopoietic lineage hierarchies and trajectories in tissues such as the human fetal liver and fetal bone marrow to not only identify both the cell of origin in which driver mutations and chromosomal alterations initially arise or are expressed but also to more fully decipher the mechanisms that subsequently promote the development of hematopoietic malignancies in the few selected individuals who will eventually be diagnosed with these diseases. For example, if the initiating mutations for clonal hematopoiesis and CHIP arise in utero and are expressed in a fetal HSC subset that is more highly proliferative and possesses higher self-renewal capacity and multipotentiality than do the HSCs of aging adult bone marrow, then these mutated clonal HSCs may have a significant competitive growth advantage over the quiescent HSCs that normally reside in post-natal and aging bone marrow. There is thus a need to more fully understand the mechanisms that provide such cells with a competitive advantage for progression to CHIP, and whether this relates to a genetic predisposition, the specific subtype of initiating mutation, further gene mutations, or differences in HSC fate determination that are controlled epigenetically, by inflammatory states or by the nutrient signaling and sensing hubs of the mitochondria and lysosomes. Of further importance has been the increasing complexity of the chromosomal alterations or mutations that have been determined by using advanced high throughput sequencing platforms and which have led to the identification and more accurate classification and risk stratification of many malignant hematopoietic subtypes. It is clear that our understanding of human hematopoiesis at each end of the age spectrum is much more limited than it is in murine model systems. A detailed analysis of the broader experimental approaches to defining the effects of aging on hematopoiesis and of the many unknowns still facing us is presented in [4] and is not be reiterated here, except to emphasize that this review provides a glimpse of some of the amazing progress already made in researching these areas of hematopoiesis over the past five decades.

## Data Availability

Not applicable.
